# The role of Vit D and parathyroid hormone in clinical severity of patients with bipolar disorder

**DOI:** 10.1192/j.eurpsy.2021.243

**Published:** 2021-08-13

**Authors:** C. Palummo, L. Marone, V. Caivano, A. Vece, L. Steardo, M. Luciano, A. Di Cerbo, V. Del Vecchio, A. Fiorillo

**Affiliations:** 1 Department Of Psychiatry, University of Campania “Luigi Vanvitelli”, Naples, Italy; 2 Health Sciences, University Magna Graecia, Catanzaro, Italy

**Keywords:** bipolar disorder, vitamin D, symptoms, calcium levels

## Abstract

**Introduction:**

Vitamin D modulates the biosynthesis of neurotransmitters and neurotrophic factors, thus influencing mood and its alterations. Decreased blood levels of Vitamin D are involved in many psychiatric disorders, in particular, affective disorders. As regards bipolar disorder (BD), an association between vitamin D deficiency and severity of illness has been found.

**Objectives:**

In this observational study, we assessed calcium homeostasis imbalance in a sample of patients with BD; in particular, we explored whether serum levels of PTH, Vitamin D and calcium influence the clinical presentation of BD and its symptom severity.

**Methods:**

All patients were administered with validated assessment instruments to assess psychopathology, affective temperaments and global functioning. Vitamin D and PTH levels were assessed in all patients. An-ad hoc schedule was administered for socio-demographic and clinical characteristics.

**Results:**

The total sample consisted of 199 patients (females: 51%; mean age: 47.1 ± 13.2 years). Levels of serum PTH were directly correlated with the total number of hospitalizations (p< 0.01), and of depressive (p< 0.0001), manic (p< 0.001) and hypomanic episodes (p< 0.01). Serum levels of Vitamin D were positively associated with age at first psychiatric contact and were inversely correlated with the total number of depressive episodes (p< 0.05) and cyclothymic temperament (p< 0.05).

**Conclusions:**

Increased levels of PTH and Vit D correlate with a worse clinical outcome of patients with BD. Our results highlight the importance to routinely assess PTH, Vit D and calcium levels in BD patients. Moreover, vitamin D may represent a valid add-on treatment for these patients.

**Disclosure:**

No significant relationships.

